# Janus and PI3-kinases mediate glucocorticoid resistance in activated chronic leukemia cells

**DOI:** 10.18632/oncotarget.11618

**Published:** 2016-08-25

**Authors:** Sina Oppermann, Avery J. Lam, Stephanie Tung, Yonghong Shi, Lindsay McCaw, Guizhei Wang, Jarkko Ylanko, Brian Leber, David Andrews, David E. Spaner

**Affiliations:** ^1^ Biology Platform, Sunnybrook Research Institute, Toronto, Ontario, Canada; ^2^ Department of Medical Biophysics, University of Toronto, Toronto, Ontario, Canada; ^3^ Department of Medicine, McMaster University, Hamilton, Ontario, Canada; ^4^ Sunnybrook Odette Cancer Center, Toronto, Ontario, Canada; ^5^ Department of Medicine, University of Toronto, Toronto, Ontario, Canada; ^6^ Department of Immunology, University of Toronto, Toronto, Ontario, Canada; ^7^ Department of Biochemistry, University of Toronto, Toronto, Ontario, Canada

**Keywords:** chronic lymphocytic leukemia, glucocorticoids, cytokines, FOXO, STAT3

## Abstract

Glucorticoids (GCs) such as dexamethasone (DEX) remain important treatments for Chronic Lymphocytic Leukemia (CLL) but the mechanisms are poorly understood and resistance is inevitable. Proliferation centers (PC) in lymph nodes and bone marrow offer protection against many cytotoxic drugs and circulating CLL cells were found to acquire resistance to DEX-mediated killing in conditions encountered in PCs including stimulation by toll-like receptor agonists and interactions with stromal cells. The resistant state was associated with impaired glucocorticoid receptor-mediated gene expression, autocrine activation of STAT3 through Janus Kinases (JAKs), and increased glycolysis. The JAK1/2 inhibitor ruxolitinib blocked STAT3-phosphorylation and partially improved DEX-mediated killing of stimulated CLL cells *in vitro* but not in CLL patients *in vivo*. An automated microscopy-based screen of a kinase inhibitor library implicated an additional protective role for the PI3K/AKT/FOXO pathway. Blocking this pathway with the glycolysis inhibitor 2-deoxyglucose (2-DG) or the PI3K-inhibitors idelalisib and buparlisib increased DEX-mediated killing but did not block STAT3-phosphorylation. Combining idelalisib or buparlisib with ruxolitinib greatly increased killing by DEX. These observations suggest that glucocorticoid resistance in CLL cells may be overcome by combining JAK and PI3K inhibitors.

## INTRODUCTION

High-dose glucocorticoids (HDGCs) are a useful treatment modality for CLL even in the era of novel agents [1,2]. HDGCs sometimes yield impressive remissions [3] but are not curative and eventually select for resistance [2]. In circulating CLL cells, glucocorticoids (GCs) activate a transcriptional program that leads to atrophy and death subsequent to decreased glycolysis from down-regulated pyruvate kinase (PK) M2 expression and function [4].

Circulating CLL cells arise from proliferation centers (PCs) in lymphoid organs and bone marrow [5]. CLL cells in these microenvironments divide in response to stimulation from signals such as antigens, costimulatory molecules, toll-like receptor (TLR) ligands, chemokines, and cytokines produced by T, nurse, stromal, and other CLL cells. These supportive microenvironments are sanctuaries that allow CLL cells to resist cytotoxic drugs [6, 7] but little is known about their effects on responses to GCs.

Surrogate models are required to study the behavior of cells in PCs as they are not readily accessible for experimental studies. Such models include coculturing CLL cells with stromal cells that express costimulatory molecules and/or activating CLL cells with mitogenic factors such as IL4 or ligands for TLRs, particularly TLR9 [8-12]. TLR9- and TLR7/8-signaling are similar and we have used the TLR-7/8 agonist resiquimod along with IL2 to mimic T cell signals in order to capture important features of PCs encountered by CLL cells *in vivo* [7, 10-12]. In this system CLL cells increase in size, proliferate, up-regulate expression of activation markers, make factors that activate the STAT3 transcription factor [13], and acquire resistance to drugs such as vincristine, fludarabine, and venetoclax [14, 15, 16]. In common with most *in vitro* PC models, the limitations include higher oxygen and glucose concentrations [17] and fewer intercellular interactions than exist *in vivo*. Nevertheless, results with this system suggest that microenvironmental signals from cytokines and phosphatidylinositol-3-kinase (PI3K) mediate resistance of CLL cells to glucocorticoids. Concomitant inhibition of both pathways may improve therapeutic outcomes with glucocorticoids in CLL.

## RESULTS

### Stimulated CLL cells resist glucocorticoid-mediated transcriptional death

**Figure 1:**
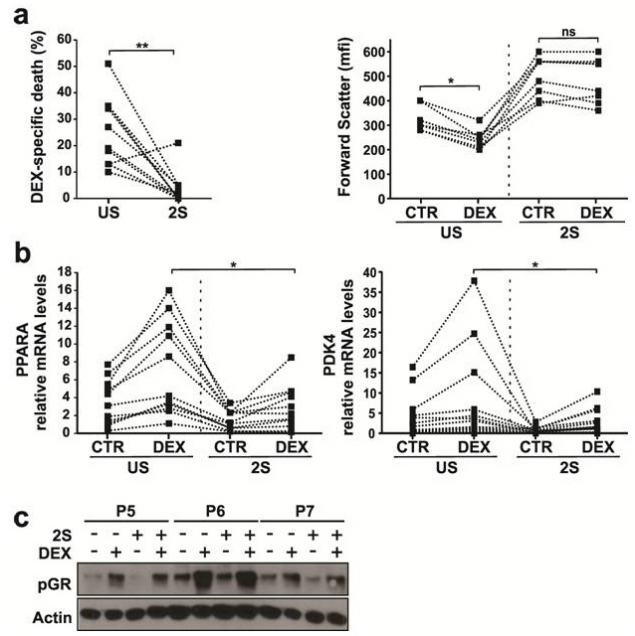
Effect of Dexamethasone on stimulated CLL cells CLL cells from 10 patients were purified and cultured in the presence (2S) or absence (unstimulated (US)) of IL-2 and resiquimod with or without (control, CTR) dexamethasone (30 μM, DEX) in serum free media. **a.** Cells were stained with 7AAD 48 h after DEX treatment and analyzed by flow cytometry. Specific death (i.e. the difference between the percentages of 7AAD^−^ cells in the control and DEX treated samples) and median forward scatter (mfi) (indicating cell size) for individual patient samples are shown on the left and right panels, respectively. **b.** mRNA levels of *PPARA* (left) and *PDK4* (right) (relative to HPRT) were determined by quantitative PCR in 20 different samples 18 h after treatment. **c.** Immunoblots of Ser211 phosphorylated glucocorticoid receptor (P-GR) in unstimulated CLL cells (−) and 2S-stimulated cells (+) with and without dexamethasone (DEX) treatment. Protein lysates for patient cells were collected 18 h after DEX treatment and immunoblots were probed with antibodies to pGR and actin used as a loading control. Representative results for patients P5, P6 and P7 are shown. *, *p* < .05; **, *p* < .01; ns, not significant.

Unstimulated CLL cells are killed by glucocorticoids such as DEX following a program of atrophy involving down-regulation of PKM2 activity and decreased glycolysis [4]. Cells can survive this death program by up-regulating expression of genes such as *PDK4* and *PPARA* and switching to fatty acid oxidation [4]. However, CLL cells stimulated with IL2 and resiquimod (2S) [11, 12, 19] are resistant to DEX-mediated killing at a dose of 30 μM that approximates plasma levels following HDGCs (Figure 1a, left panel) [2, 4]. While unstimulated CLL cells atrophied and became smaller, 2S-stimulated CLL cells were larger and did not shrink significantly in response to DEX (Figure 1a, right panel). Furthermore 2S-stimulated CLL cells did not increase their expression of *PPARA* and *PDK4* mRNA transcripts, which are up-regulated in DEX-treated unstimulated cells (US) (Figure 1b).

The failure of DEX to kill 2S-stimulated cells could reflect altered glucocorticoid receptor (GR) expression or function. Ligand binding leads to phosphorylation and release of cytoplasmic GRs from heat shock proteins with subsequent entry into the nucleus to mediate gene transcription [4]. GR phosphorylation after DEX was not much different in US and 2S-stimulated cells, suggesting the initial steps of the response were intact (Figure 1c).

### JAK inhibitors sensitize activated CLL cells to DEX *in vitro* but not *in vivo*

Cytokine signaling through Janus kinases (JAKs) inhibits transcriptional activity of the glucocorticoid receptor [20, 21] and 2S-stimulated cells produce a number of cytokines, including IL10 and TNF-α [12]. These observations suggested JAK inhibitors might increase DEX-mediated killing of 2S-stimulated CLL cells.

**Figure 2:**
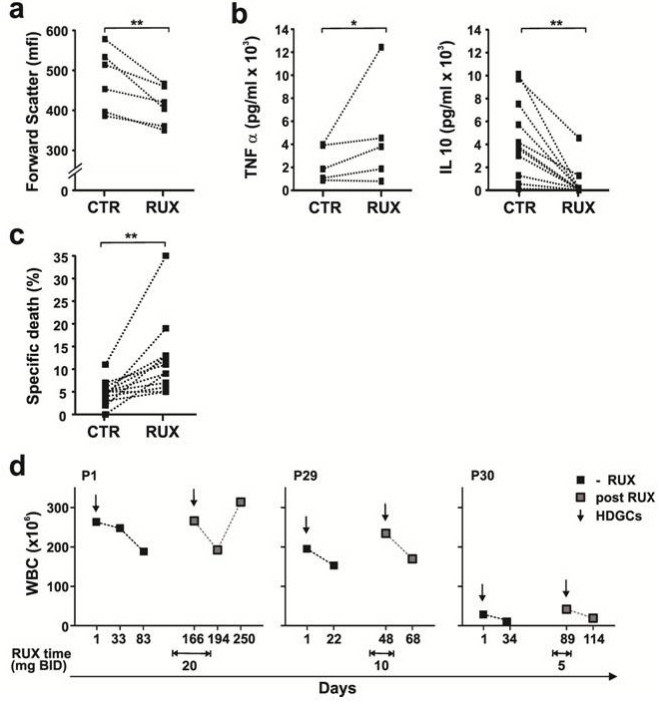
Effect of Ruxolitinib on DEX-mediated death in stimulated CLL cells *in vitro* and *in vivo* Purified CLL cells were stimulated with IL2 and Resiquimod in the presence or absence of dexamethasone (30 μM) or ruxolitinib (0.5 μM). **a.** Size changes of viable 7AAD- cells (median of forward scatter parameter (mfi)) were determined by flow cytometry after 48 h. Decreased size due to activation in the presence of ruxolitinib (RUX) is indicated for each patient sample by the lines. **b.** After 48 h, levels of TNFα (left panel, *n* = 5) and IL10 (right panel, *n* = 11) in the culture supernatants of 2S cells in the presence and absence (CTR) of RUX were measured by ELISAs. **c.** After 48 h, specific death was calculated from the difference between the percentages of 7AAD- cells in control and DEX-treated samples 48 h after RUX treatment (*n* = 12). **d.** Changes in circulating lymphocytes (WBC) as a measure of clinical response are shown for 3 patients 1 month after treatment with high-dose glucocorticoids in the presence or absence of Ruxolitinib at the doses indicated in the graphs. *, *p* < .05; **, *p* < .01.

Ruxolitinib is a selective inhibitor of JAK1 and JAK2 that is licensed to treat myelofibrosis [22] and being evaluated in CLL [23]. Ruxolitinib decreased cell size and IL10 production and increased TNFα production by 2S-stimulated CLL cells *in vitro* (Figure 2a and 2b). Consistent with a role for STAT3 and IL10 in promoting glucocorticoid-resistance, ruxolitinib significantly increased the killing of 2S-stimulated cells by DEX (Figure 2c).

A clinical trial of ruxolitinib as first-line treatment for CLL patients [23] provided an opportunity to assess the effect of this drug on glucocorticoid responses *in vivo*. Three subjects in this trial had been treated with HDGCs (187.5 mg/m^2^ DEX for 4 days) several months before commencing ruxolitinib and required another course of HDGCs on ruxolitinib to treat cytopenias. Ruxolitinib was administered for all 4 days of HDGCs in patient 1 but the overlap was 1 day in patients 29 and 30 as ruxolitinib was held following initiation of DEX (Figure 2d). Circulating lymphocyte counts (WBC) prior to HDGCs and 4 weeks later were used to measure clinical response. The decrease in circulating lymphocyte counts after HDGCs was not enhanced by ruxolitinib, suggesting CLL cells had not been sensitized *in vivo* (Figure 2d).

### Identification of PI3K/AKT inhibitors as glucocorticoid-sensitizing agents by automated high-content confocal fluorescent microscopy

**Figure 3:**
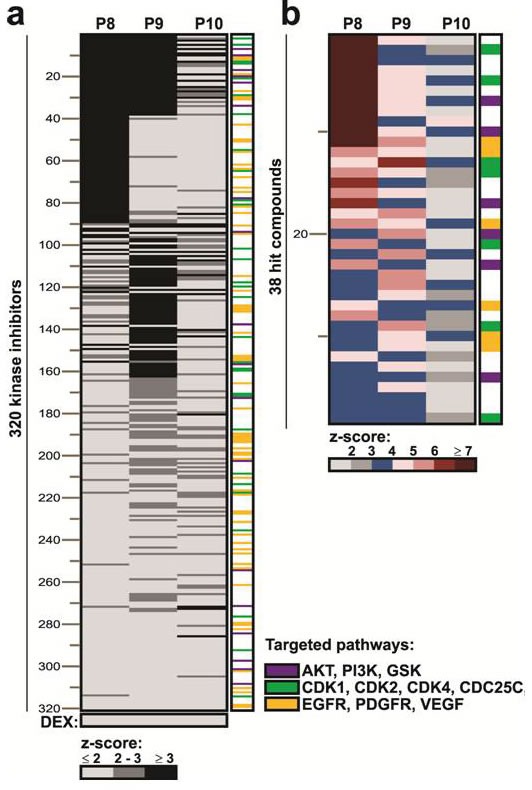
Heatmap for DEX-enhanced drug activity in 3 patients **a.** Three hundred twenty different kinase inhibitors (KI) were screened for DEX-enhanced toxicity against purified 2S-stimulated CLL cells from 3 patients (P8, P9, P10) using TMRE fluorescence as an indicator of mitochondrial potential and cell death. Individual patient samples are shown in columns and 320 KIs are indicated in rows. Compound activity for the synergy screen is indicated by positive z-scores for each sample as indicated by the grey scale bare below. Kinase inhibitor and DEX combination were scored as positive if z-scores were ≥ 3 (black). Compounds are ranked by average of z-score> 3 with highest z-score on top and lowest z-score at the bottom. Common pathways targeted by the kinase inhibitors are indicated by color in the last column and listed on the right. **b.** Magnified version of the full heat-map, showing the top 38 kinase inhibitor hits. Color scale bar indicates drug efficacy by increase in z-score. The names of all kinase inhibitors are provided in supplementary Table S2.

Other signaling pathways activated in CLL cells following stimulation with IL2 and resiquimod might mediate resistance to glucocorticoids and insensitivity to JAK inhibitors. To identify these pathways, an unbiased high-content image-based microscopic approach [15, 24] was used to screen a library of 320 kinase inhibitors (KIs) (Supplementary Table S2) on 2S-stimulated CLL cells from three different patients. The KIs (1 μM) were evaluated alone and in combination with DEX (30 μM) (Figure 3a) with the hypothesis that common hits that enhanced DEX-mediated death should reveal the protective pathways. Cell stress and death were assessed by automated image analysis of 2 fluorescent channels. Cells were stained with a red lipophilic TMRE dye that measures loss of mitochondrial membrane potential in dying cells and Draq5 (blue) to obtain total cell counts. Drug responses were assessed by applying intensity and area thresholds established from DMSO treated controls [15l]. KI efficacy was assessed by z-score analysis of the TMRE signal and compounds with a z-score ≥ 3 were identified as hits for DEX-enhanced kill (i.e. an increase in dead cells above KI treatment alone). Stimulated cells from all three patients were resistant to DEX (Figure 3a, bottom) and exhibited variable responses to DEX plus KI treatment (Figure 3a). Compounds were ranked by average z-score across all 3 patients, and the most common targeted pathways indicated by a color scheme (Figure 3, bottom). To identify the most important pathways that mediated glucocorticoid resistance, the top 38 KIs with z-scores ≥ 3 for at least 2/3 patients were further distinguished from compounds with z-scores from ≤ 2 to ≥7 as shown in the magnification heatmap (Figure 3b, Supplementary Table S2).

A JAK inhibitor was among the top 38 KIs, but the major classes of inhibitors that sensitized 2S cells to DEX were inhibitors of receptor tyrosine kinases (EGFR, PDGFR, VEGFR, 6/38 inhibitors, orange), cell cycle regulators (7/38 inhibitors, green), and members of the PI3K/AKT/GSK3 pathway (8/38 inhibitors, purple) (Figure 3b). The high-frequencies of these pathways could not be explained simply by the proportion of pathway-specific inhibitors in the library. While compounds targeting the first two pathways are not used presently to treat CLL, the latter pathway is an important target [25] and was chosen for more detailed studies.

### Inhibition of glycolysis restores glucocorticoid-sensitivity in stimulated CLL cells

**Figure 4:**
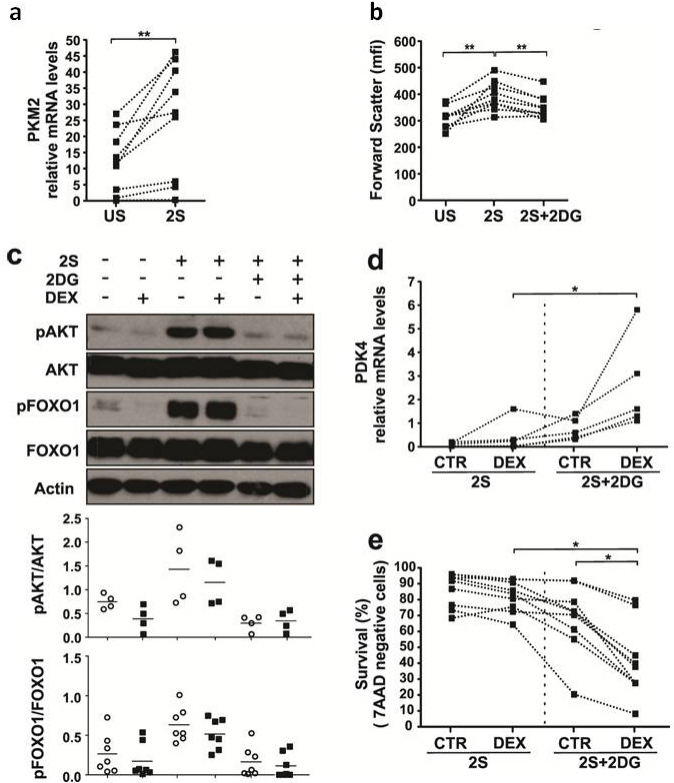
Effect of 2-deoxyglucose on sensitivity of 2S-stimulated CLL cells to dexamethasone CLL cells were purified and cultured in the presence (2S) or absence (unstimulated (US)) of IL-2 and resiquimod with or without dexamethasone (DEX) (30 μM) and 2-deoxyglucose (2DG) (3mM). After 18 h, *PKM2* **a.** and *PDK4* **d.** transcripts (relative to HPRT) were measured by quantitative PCR. The lines indicate results obtained from samples from the same patient. **b.** After 48 h, flow cytometry was used to determine the size of viable 7AAD^−^ cells by the median of the forward scatter parameter (mfi). **c.** After 18 h, phosphorylated and un-phosphorylated FOXO1 and AKT levels were measured by immunoblotting and quantified by densitometry. Circles indicate absence and black squares indicated presence of DEX treatment. An example is shown and relative densitometric values are plotted in the lower graphs, with results for individual patient samples (*n* = 4 for AKT and *n* = 7 for FOXO1). **e.** Percentages of viable 7AAD^−^ cells that exclude 7AAD were determined by flow cytometry after 48h. *, *p* < .05; **, *p* < .01.

Activation of the PI3K/AKT pathway is associated with increased glucose transport and glycolysis [26] and marked by phosphorylation of substrate FOXO proteins [27]. Consistent with increased glycolysis, PKM2 transcript levels were increased in 2S-stimulated cells (Figure 4a). Inhibition of AKT can be achieved by inhibiting glycolysis with 2-deoxyglucose (2-DG) [28] and sensitizes acute leukemia cells to DEX [29, 30]. 2-DG (3 mM) decreased the size of 2S-stimulated CLL cells and their expression of phospho-AKT and -FOXO1 (Figure 4b, 4c). DEX-mediated induction of *PDK4* mRNA (Figure 4d) was restored by 2-DG and associated with enhanced killing (Figure 4e).

Based on these results, 2-DG should improve glucocorticoid-efficacy in CLL patients but it is unfortunately too toxic for clinical use [31]. The AKT pathway can also be targeted by clinically relevant PI3K inhibitors such as idelalisib (IDE), a specific inhibitor of the PI3Kδ isozyme, and buparlisib (BKM), a pan-class I PI3K inhibitor [25, 32, 33, 34]. These agents were evaluated for their ability to sensitize 2S-stimulated CLL cells to DEX.

### PI3K inhibitors combined with ruxolitinib enhance DEX-mediated death in stimulated CLL cells

**Figure 5:**
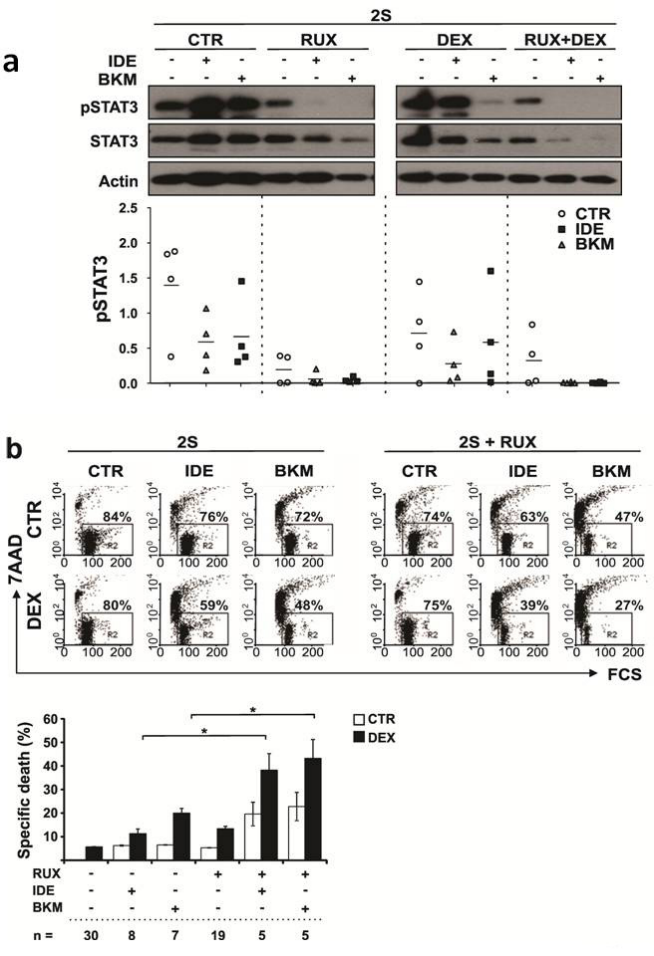
Effect of PI3K inhibitors and ruxolitinib on phospho-STAT3 levels and DEX-mediated death of activated CLL cells **a.** 2S CLL cells were stimulated with IL2 and resiquimod and treated with or without dexamethasone (DEX) (30 μM) or ruxolitinib (RUX) or the combination of DEX and RUX, each in the presence and absence of idelalisib (IDE) or buparlisib (BKM). Protein lysates of 2S-stimulated cells were collected for immunoblotting 18 h after the treatments. A representative example of immunoblots for pSTAT3 and STAT3 is shown at the top of the graph. Results for pSTAT3 for 4 different patient samples were quantified by densitometry and normalized to actin as shown in the bottom panel. The horizontal lines represent mean values for the 4 samples. **b.** An example of flow cytometric analysis of cell death in 2S-stimulated cells in the absence (left) and presence (right) of ruxolitinib (2S+RUX) assessed by 7AAD staining is shown in the top dot-plots. The upper row (CTR) shows single agent treatment with IDE or BKM (0.5 μM each) while the lower row indicates respective treatments in the presence of DEX. The numbers in each dot-plot represent percentages of viable 7AAD^−^ cells. Averages and standard errors for the indicated numbers of patient samples are shown in the bottom panel. *, *p* < 0.05.

Single agent buparlisib and idelalisib were not toxic to 2S-stimulated CLL cells at concentrations up to 3 μM (Supplementary Figure 1). A dose of 0.5 μM, below plasma concentrations obtained with these drugs [32, 33], was chosen for further studies and found to increase DEX-mediated killing of 2S-stimulated cells (Figure 5b; left 3 panels). However, the increases, albeit significant, were of the order of 10-20% and similar to those obtained with ruxolitinib *in vitro* that proved not to have therapeutic efficacy *in vivo* (Figure 2c, 2d). These observations suggested a need to inhibit additional compensatory signaling pathways and achieve greater killing *in vitro* to expect to overcome glucocorticoid resistance in CLL cells in patients [35].

To identify signaling pathways that remained active in the presence of PI3K inhibitors, 2S-stimulated CLL cells from four patients were treated with idelalisib or buparlisib, analyzed with a phosphokinase array (not shown), and the results confirmed by immunoblotting (Figure 5a). Regardless of the presence of DEX, phospho-STAT3 levels were found to remain high in the face of PI3K inhibition (Figure 5a). The effect of adding ruxolitinib was then evaluated due to its ability to inhibit JAK-mediated STAT3 activation [23]. Treatment with both ruxolitinib and burparlisib or idelalisib eliminated pSTAT3 almost entirely, especially in the presence of DEX (Figure 5a). Importantly, DEX-mediated death was enhanced considerably when either PI3K inhibitor was used together with ruxolitinib (Figure 5b, right 3 panels and lower panel). High doses of the PI3K inhibitors (3 μM) and ruxolitinib (1 μM) proved directly toxic to 2S-stimulated cells (Supplementary Figure 1).

### Combining kinase inhibitors overcomes glucocorticoid resistance in CLL cells cocultured with CD40L-expressing stromal cells

**Figure 6:**
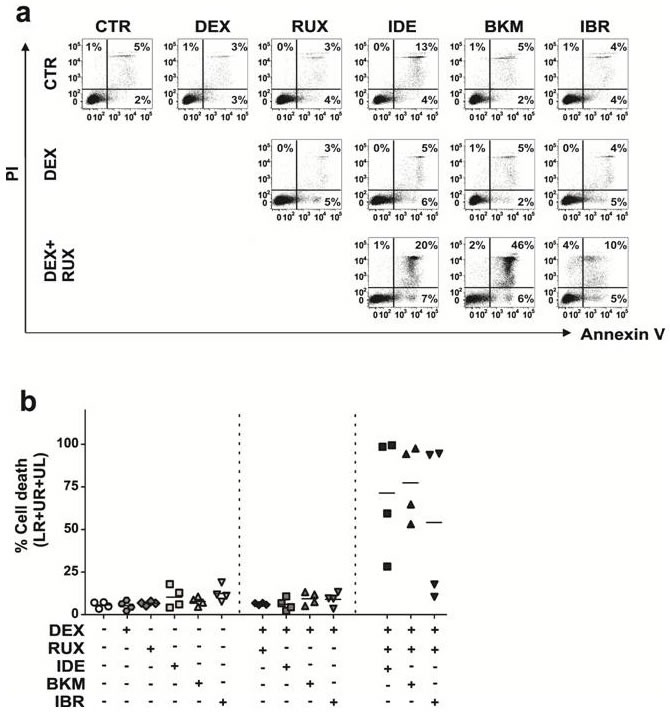
PI3K inhibitors combined with Ruxolitinib overcome resistance to DEX in CLL cells cocultured with OP9-CD40L expressing stromal cells CLL cells from 4 different patients were co-cultured with OP9 stromal cells expressing human CD40L (CD40L) and treated with idelalisib (IDE, 3 μM), buparlisib (BKM, 3 μM) or ibrutinib (IBR, 3 μM) alone, in combination with dexamethasone (DEX, 30 μM), and with both DEX and ruxolitinib (RUX, 1 μM). Drug response was determined after 48 h by flow cytometric analysis of Annexin V (AV) and propidium iodide (PI) stained CLL cells. **a.** Dot blots are shown for one representative patient. The numbers in the dot blots indicate percentage of AV+ (LR), AV+PI+ (UR) and PI+ (UL) cells. **b.** Summary graph showing percentages of dead cells (sum of AV+, AV+/PI+ and PI+ cells) for CD40L-stimulated cells from 4 patients. The lines indicate mean values for percentages of dead cells (*n* = 4).

These results, obtained in a specific *in vitro* model using 2S-stimulated CLL cells, suggested that PI3K inhibitors combined with ruxolitinib might be an effective strategy for overcoming glucocorticoid-resistance in CLL microenvironments. To show the results were not simply model-dependent, the experiments were repeated in a stromal cell coculture model. CLL cells from four patients were cocultured with murine bone marrow stromal cells (OP9) expressing human CD40L [9, 15, 36, 37, 38]. Consistent with results from 2S-stimulated cells, cocultured CLL cells acquired resistance to DEX (30 μM) and were not killed by ruxolitinib, buparlisib or idelalisib alone (Figure 6). Neither buparlisib nor idelalisib reversed DEX-resistance (average kill of 9% and 6%, respectively, Figure 6a and 6b). However, combining the PI3K inhibitors with ruxolitinib strikingly increased DEX-mediated cell death (average cell kill of 77% and 71% for buparlisib and idelalisib, respectively (Figure 6a and 6b, *n* = 4)), confirming the importance of blocking multiple kinase-dependent pathways.

### Effect of ibrutinib on glucocorticoid-resistance

The Burton's tyrosine kinase (BTK) inhibitor ibrutinib was not included in the KI library used for screening (Supplementary Table S2). Based on its importance in the clinical management of CLL [1] and ability to inhibit AKT-signaling indirectly [39], studies were carried out to determine if ibrutinib might also combine with ruxolitinib to sensitize resistant CLL cells to glucocorticoids.

In the 2S model, no toxicity was seen with concentrations of ibrutinib alone up to 3 μM. In contrast to buparlisib and idelalisib, no toxicity occurred when 3 μM of ibrutinib was combined with ruxolitinib (Supplemental Figure 1).

In the OP9-CD40L stromal cell coculture model, ibrutinib had minimal effects on cell death, either as a single agent or in combination with DEX (Figure 6) (average cell death of 12% and 9% respectively, *n* = 4). Combining ibrutinib and ruxolitinib enhanced DEX-mediated death in 2 out of 4 patients (average total kill of 54% for all four patients (Figure 6)).

## DISCUSSION

The studies in this manuscript provide insights into why glucocorticoids are only partially effective in treating CLL. They suggest that signals from the PC microenvironment prevent glucocorticoids from initiating cell death transcriptional programs in CLL cells (Figure 1). These signals appear to involve multiple pathways. Cytokine-signaling through Janus Kinases is one important mediator of glucocorticoid-resistance (Figure 2). Our high-throughput screen of more than 300 kinase inhibitors also identified PI3K/ATK, receptor tyrosine kinases and cell cycle regulators as potential targets (Figure 3). Taken together, the observations suggest that blocking protective signals from the microenvironment with kinase inhibitors may be a useful strategy to improve the therapeutic efficacy of glucocorticoids in CLL. However, combinations of kinase inhibitors are needed to deal with the complexity of the protective signals (Figure 5, 6).

The precise mechanisms that cause stimulated CLL cells to become resistant to DEX are not known and may be specific for individual patients. While there was some variability in resistance of 2S-stimulated CLL cells to DEX-mediated killing, it did not correlate with the clinical characteristics of the patients (Supplementary Table S1). There was also some variability in the responses of 2S-stimulated CLL cells to different kinase inhibitors (Figure 3). Combining PI3K and JAK inhibitors is expected to improve the outcomes of most CLL patients who require high-dose glucocorticoids but there may be some who will benefit from unique kinase inhibitors. High-content microscopy-based screening of kinase inhibitor libraries, as described here (Figure 3) and elsewhere [15], may potentially identify such inhibitors.

The initial steps of glucocorticoid signaling are intact in stimulated CLL cells (Figure 1c) and the explanation for glucocorticoid-resistance appears to lie further downstream at the transcriptional level [40]. Promoter regions of genes that are subject to regulation by glucocorticoids are often complex and the outcome may be affected significantly by cooperating transcription factors such as members of the FOXO and PPAR gene families [41]. Activated signaling pathways in stimulated CLL cells affect the localization or expression of these factors [42], preventing glucocorticoids from mediating the transcriptional responses responsible for cellular atrophy and death [4]. For example, PPARα is significantly down-regulated in stimulated CLL cells [11] and phosphorylation prevents FOXO1 from entering the nucleus to support transcriptional programs with the GR [27]. The association of high-levels of pFOXO1 in stimulated DEX-resistant CLL cells and its absence in DEX-sensitive cells (Figure 4c) is consistent with a role in glucocorticoid-mediated resistance of CLL cells as in other cell-types [42, 43]. The role of cytokines (Figure 2b) and receptor tyrosine kinases (Figure 3, yellow) may be linked to their ability to activate the PI3K/AKT pathway (Figure 3, 5, 6) as well as drive CLL cells through the cell-cycle [12]. Cell cycle proteins (Figure 3, green) have also been shown to negatively regulate transcription by nuclear receptors [44].

Despite increased killing of stimulated CLL cells *in vitro* and prevention of STAT3-phosphorylation in response to exogenous cytokines *in vitro* (Figure 5), clinical responses to HDGCs were not noticeably improved by ruxolitinib (Figure 2d). A number of reasons may account for the apparent inability of single-agent ruxolitinib to increase therapeutic efficacy of HDGCs *in vivo*. The sensitizing effects ascribed to ruxolitinib were based on a specific *in vitro* microenvironmental model (Figure 1) and used a 10% increase in DEX-mediated killing as evidence for potential clinical importance (Figure 2c). Absence of sensitization to glucocorticoids *in vivo* may reflect the limitations of this model or that more *in vitro* activity (eg. as seen with ruxolitinib and PI3K inhibitor combinations (Figure 5, 6) is needed to correlate with clinical significance. Additional signaling pathways that are not inhibited by ruxolitinib but affect glucocorticoid-mediated gene transcription may also be active *in vivo*. Ruxolitinib is selective for JAKs and does not inhibit other pathways such as PI3K/AKT/FOXO. The negative clinical observations seen with ruxolitinib and DEX (Figure 2c) may reflect ongoing activation of this pathway *in vivo* preventing therapeutic glucocorticoid-mediated transcriptional responses (Figure 5, 6) [43].

Combined use of PI3K inhibitors with ruxolitinib to potentiate therapeutic activity of HDGCs in CLL is supported by the experiments in Figures 5 and 6. Information about the potential toxicity of combining buparlisib and ruxolitinib will be obtained from on-going clinical trials in myelofibrosis (NCT01730248) [22]. The selective PI3Kδ-inhibitor idelalisib may be less toxic than buparlisib due to the restricted expression of this isozyme. Either of these combinations may improve the therapeutic efficacy of glucocorticoids for most patients. Individual drug screening may optimize the identification of effective combinations for others (Figure 3) [15].

## MATERIALS AND METHODS

### CLL patients and cells

CLL cells were isolated from patients who were otherwise untreated for at least 3 months [4, 15, 18]. Clinical characteristics of the patients are shown in Supplementary Table S1. Protocols were approved by the Sunnybrook Ethics Review Board. The clinical trial of ruxolitinib [23] was registered as NCT02015208 and the high-dose glucocorticoid regimen administered to some patients consisted of oral dexamethasone (187.5 mg/m^2^) for 4 days [2].

### Antibodies and reagents

7-aminoactinomycin D (7-AAD) was obtained from Pharmingen (San Francisco, CA). Fatty acid free bovine serum albumin, 2-mercaptoethanol (2-ME), dimethyl sulfoxide (DMSO), staurosporine, human IL-10 and anti-IL10 antibodies, 2-deoxyglucose, human IL-6, and β-actin antibodies were from Sigma (St. Louis, MO). RPMI-1640 and lipid-rich bovine albumin (AlbuMAXÒII) were from Invitrogen (Carlsbad, CA). Resiquimod (TLR-7/8 agonist) was from Alexis Biochemicals (San Diego, CA). Clinical grade dexamethasone sodium phosphate (DEX) (Omega, Montreal, Quebec) and IL2 (Novartis Pharmaceuticals Canada Inc., Dorval, Quebec) were purchased from the hospital pharmacy. Ruxolitinib, idelalisib, and buparlisib and ibrutinib were purchased from Selleck Chemicals (Houston, TX). The fluorescent dye tetramethylrhodamine ethyl ester (TMRE) was purchased from Life Technologies (Carlsbad, California, USA) and Draq5 was from Biostatus (Leicestershire, UK). Antibodies against phospho-(Ser^211^)glucocorticoid receptor, STAT3, AKT, FOXO1, phospho-(Tyr^705^)STAT3, phospho-(Thr^308^)AKT, and phospho-(Ser^256^)FOXO1 were from Cell Signaling Technology (Beverly, MA), as were secondary horseradish peroxidase-conjugated anti-rabbit and anti-mouse antibodies (Cat. Nos. 7074 and 7076, respectively). The GSK published kinase inhibitor set (PKIS) was a gift of Dr. Bill Zuercher (GSK). Human phospho-kinase array kits (#ARY003B) were from R&D (Minneapolis, MN) and used accordingly to the manufacturer's instructions and website.

### Cell culture

Unless otherwise specified, purified CLL cells were cultured at a concentration of 1 × 10^6^ cells/mL in RPMI-1640 medium supplemented with Transferrin and 0.02% AlbuMAX II in 6- or 24-well plates (BD Labware) at 37°C in 5% CO_2_ for the times indicated. Resiquimod and IL2 were used at 1 μg/ml and 500 U/ml. CLL cells stimulated in this manner are designated “2S” cells [11, 12]. OP9-CD40L cells were generated and cultured in alpha-MEM supplemented with 20% FBS (Gibco) as described before [15].

### CLL-OP9 coculture experiments

CD40L-expressing OP9 bone marrow stromal cells were seeded into 24-well plates at a concentration of 5×10^4^ cells/well the day before initiation of CLL cocultures. CLL cells were added onto the stromal layer at a final concentration of 1×10^5^ cells/ml. For comparison, suspension cells of the same CLL sample were cultured at a density of 1×10^5^ cells/ml. Coculture and suspension cells were treated with dexamethasone (30 μM, DEX), ruxolitinib (1 μM, RUX), ibrutinib (3 μM, IBR), idelalisib (3 μM, IDE) or buparlisib (3 μM, BKM) alone or in combination as indicated. Cells were collected by pipetting after 48 h, leaving the intact adherent stromal layer behind.

### Plasma inhibitor assay for ruxolitinib levels

Plasma samples were obtained before each cycle of ruxolitinib and stored at −80°C. To determine if plasma levels of ruxolitinib were sufficient to inhibit IL6-mediated STAT3-phosphorylation, 2×10^6^ cells were incubated with 1 ml of thawed plasma for 30 min and then stimulated with IL6 (40 ng/ml). Protein extracts were collected after 30 min and subjected to immunoblotting.

### Flow cytometry

Cell viability was determined by staining cells with 3.5 mL of 7AAD for 10 min and washing in phosphate-buffered saline (PBS). Flow cytometric detection of Annexin V and PI stained cells was performed using the Alexa Fluor 488 Annexin V/dead cell apoptotis kit (Thermofisher Scientific) according to the manufacture's protocol. Cells were co-stained with Alexa Fluor 488 Annexin V and propidium iodide (PI). Flow cytometry was performed using the BD LSRII flow cytometer. Annexin V and PI were excited using the 488 nm blue laser and 505 LP filter and the 545 nm laser and 635 LP filter, respectively. Twenty-five thousand counts were measured and data analyzed using Flowjo 9.6 software (Tree Star, San Carlos, CA).

### Immunoblotting

Protein extraction and immunoblotting were performed as previously described [18].

### Real-time PCR

RNA was prepared with the RNeasy mini kit (Qiagen, Valencia, CA, USA) and cDNA was synthesized from 2 g of RNA using Superscript III reverse transcriptase (Life Technologies, Invitrogen), according to the manufacturer's instructions. The following primers were used: PKM2 forward, GTCGAAGCCCCATAGTGAAG; reverse, ATGTCCTTCTCCGACACAGC, PPARA forward, CTGGAAGCTTTGGCTTTACG; reverse, ACCAGCTTGAGTCGAATCGT, PDK4 forward, CATACTCCACTGCACCAACG; reverse, CCTGCTTGGGATACACCAGT, and HPRT forward, GAGGATTTGGAAAGGGTGTT; reverse ACAATAGCTCTTCAGTCTGA. Polymerase chain reactions were carried out in a DNA engine Opticon System (MJ Research, Waltham, MA, USA) and cycled 34 times after denaturation (95°C, 15 min) with the following parameters: denaturation at 94°C for 20 sec, annealing at 58°C for 20 sec, and extension at 72°C for 20 sec. mRNA abundance was evaluated by a standard amplification curve. Copy numbers were determined from two independent cDNA preparations for each sample and expressed as the relative fold change of the target to HPRT.

### Cytokine measurements

Cytokine levels in culture supernatants were measured with commercial ELISA kits for human IL10 and TNFα according to the manufacturer's instructions (R&D Systems, Minneapolis, MN).

### High content kinase inhibitor screening

CLL cells were seeded at 1.5-2×10^6^ cells/ml with IL-2 and resiquimod in a 384-well plate (Cell Carrier; PerkinElmer, Waltham, MA) and cultured at 37^ο^ C and 5% CO_2_. Individual members of the kinase inhibitor library were dissolved in DMSO and added to the cultures at final concentrations of 1 μM. After 72 h, fluorescent dyes were added for 30 min prior to image acquisition. Tetramethylrhodamine ethyl ester (TMRE; Life Technologies) at 10 nM and Draq5 (Biostatus, Leicestershire, UK) at 5 μM were used to assess mitochondrial membrane potential and detect cell nuclei for cell segmentation, respectively.

### High content confocal fluorescent microscopy and image analysis

Confocal microscopy was performed at 37°C and 5% CO_2_ using an Opera QEHS high-content screening system (PerkinElmer) as described [15]. TMRE intensity thresholds were established for each sample from untreated control cells compared to a positive control for dead cells treated with staurosporin.

Based on the TMRE signal, drug combinations that enhanced DEX-mediated cell death were identified by z-score analysis using the mean and standard deviation of eight in-plate DMSO control replicate wells. Enhanced DEX-mediated cell kill was calculated by the total cell kill induced by the combination of DEX and KI above cell kill induced by kinase inhibitors alone. Kinase inhibitors with z-scores ≥3 were scored as positive hits.

### Statistical analysis

Student's *t*-test and paired t-tests were used to determine *p*-values.

## SUPPLEMENTARY MATERIALS FIGURE AND TABLES





## References

[1] Awan FT, Byrd JC (2014). New strategies in chronic lymphocytic leukemia: shifting treatment paradigms. Clin Cancer Res.

[2] Spaner DE (2012). Oral high-dose glucocorticoids and ofatumumab in fludarabine-resistant chronic lymphocytic leukemia. Leukemia.

[3] Thornton PD, Matutes E, Bosanquet AG, Lakhani AK, Grech H, Ropner JE, Joshi R, Mackie PH, Douglas ID, Bowcock SJ, Catovsky D (2003). High dose methylprednisolone can induce remissions in CLL patients with p53 abnormalities. Ann Hematol.

[4] Tung S, Shi Y, Wong K, Zhu F, Gorczynski R, Laister RC, Minden M, Blechert AK, Genzel Y, Reichl U, Spaner DE (2013). PPARalpha and fatty acid oxidation mediate glucocorticoid resistance in chronic lymphocytic leukemia. Blood.

[5] Ponzoni M, Doglioni C, Caligaris-Cappio F (2011). Chronic lymphocytic leukemia: the pathologist's view of lymph node microenvironment. Semin Diagn Pathol.

[6] Burger JA, Ghia P, Rosenwald A, Caligaris-Cappio F (2009). The microenvironment in mature B-cell malignancies: a target for new treatment strategies. Blood.

[7] Li Y, Shi Y, McCaw L, Li YJ, Zhu F, Gorczynski R, Duncan GS, Yang B, Ben-David Y, Spaner DE (2015). Microenvironmental interleukin-6 suppresses toll-like receptor signaling in human leukemia cells through miR-17/19A. Blood.

[8] Pascutti MF, Jak M, Tromp JM, Derks IA, Remmerswaal EB, Thijssen R, van Attekum MH, van Bochove GG, Luijks DM, Pals ST, van Lier RA, Kater AP (2013). IL-21 and CD40L signals from autologous T cells can induce antigen-independent proliferation of CLL cells. Blood.

[9] Purroy N, Abrisqueta P, Carabia J, Carpio C, Calpe E, Palacio C, Castellví J, Crespo M, Bosch F (2014). Targeting the proliferative and chemoresistant compartment in chronic lymphocytic leukemia by inhibiting survivin protein. Leukemia.

[10] Spaner DE, Shi Y, White D, Mena J, Hammond C, Tomic J, He L, Tomai MA, Miller RL, Booth J, Radvanyi L (2006). Immunomodulatory effects of Toll-like receptor-7 activation on chronic lymphocytic leukemia cells. Leukemia.

[11] Spaner DE, Lee E, Shi Y, Wen F, Li Y, Tung S, McCaw L, Wong K, Gary-Gouy H, Dalloul A, Ceddia R, Gorzcynski R (2013). PPAR-alpha is a therapeutic target for chronic lymphocytic leukemia. Leukemia.

[12] Tomic J, White D, Shi Y, Mena J, Hammond C, He L, Miller RL, Spaner DE (2006). Sensitization of IL-2 signaling through TLR-7 enhances B lymphoma cell immunogenicity. J Immunol.

[13] Levidou G, Sachanas S, Pangalis GA, Kalpadakis C, Yiakoumis X, Moschogiannis M, Sepsa A, Lakiotaki E, Milionis V, Kyrtsonis MC, Vassilakopoulos TP, Tsirkinidis P, Kontopidou F (2014). Immunohistochemical analysis of IL-6, IL-8/CXCR2 axis, Tyr p-STAT-3, and SOCS-3 in lymph nodes from patients with chronic lymphocytic leukemia: correlation between microvascular characteristics and prognostic significance. Biomed Res Int.

[14] Herishanu Y, Pérez-Galán P, Liu D, Biancotto A, Pittaluga S, Vire B, Gibellini F, Njuguna N, Lee E, Stennett L, Raghavachari N, Liu P, McCoy JP (2011). The lymph node microenvironment promotes B-cell receptor signaling, NF-kappaB activation, and tumor proliferation in chronic lymphocytic leukemia. Blood.

[15] Oppermann S, Ylanko J, Shi Y, Hariharan S, Oakes CC, Brauer PM, Zúñiga-Pflücker JC, Leber B, Spaner DE, Andrews DW (2016). Identification of kinase inhibitors that overcome venetoclax resistance in activated CLL cells by high-content screening. Blood.

[16] Shi Y, White D, He L, Miller RL, Spaner DE (2007). Toll-like receptor-7 tolerizes malignant B cells and enhances killing by cytotoxic agents. Cancer Res.

[17] Spencer JA, Ferraro F, Roussakis E, Klein A, Wu J, Runnels JM, Zaher W, Mortensen LJ, Alt C, Turcotte R, Yusuf R, Côté D, Vinogradov SA (2014). Direct measurement of local oxygen concentration in the bone marrow of live animals. Nature.

[18] Tomic J, Lichty B, Spaner DE (2011). Aberrant interferon-signaling is associated with aggressive chronic lymphocytic leukemia. Blood.

[19] Crassini K, Stevenson WS, Mulligan SP, Best OG (2015). The MEK1/2 inhibitor, MEKi-1, induces cell death in Chronic Lymphocytic Leukemia cells under conditions that mimic the tumor microenvironment and is synergistic with fludarabine. Leuk Lymphoma.

[20] Rider CF, Shah S, Miller-Larsson A, Giembycz MA, Newton R (2015). Cytokine-induced loss of glucocorticoid function: effect of kinase inhibitors, long-acting beta(2)-adrenoceptor [corrected] agonist and glucocorticoid receptor ligands. PLoS One.

[21] Xu Q, Goleva E, Ou LS, Li LB, Leung DY (2004). CD56+ cells induce steroid resistance in B cells exposed to IL-15. J Immunol.

[22] Stein BL, Swords R, Hochhaus A, Giles F (2014). Novel myelofibrosis treatment strategies: potential partners for combination therapies. Leukemia.

[23] Spaner DE, Wang G, McCaw L, Li Y, Disperati P, Cussen MA, Shi Y (2016). Activity of the Janus kinase inhibitor ruxolitinib in chronic lymphocytic leukemia: results of a phase II trial. Haematologica.

[24] Bullen A (2008). Microscopic imaging techniques for drug discovery. Nat Rev Drug Discov.

[25] Fruman DA, Cantley LC (2014). Idelalisib-a PI3Kdelta inhibitor for B-cell cancers. N Engl J Med.

[26] Elstrom RL, Bauer DE, Buzzai M, Karnauskas R, Harris MH, Plas DR, Zhuang H, Cinalli RM, Alavi A, Rudin CM, Thompson CB (2004). Akt stimulates aerobic glycolysis in cancer cells. Cancer Res.

[27] Hedrick SM (2009). The cunning little vixen: Foxo and the cycle of life and death. Nat Immunol.

[28] Tzatsos A, Tsichlis PN (2007). Energy depletion inhibits phosphatidylinositol 3-kinase/Akt signaling and induces apoptosis via AMP-activated protein kinase-dependent phosphorylation of IRS-1 at Ser-794. J Biol Chem.

[29] Hulleman E, Kazemier KM, Holleman A, VanderWeele DJ, Rudin CM, Broekhuis MJ, Evans WE, Pieters R, Den Boer ML (2009). Inhibition of glycolysis modulates prednisolone resistance in acute lymphoblastic leukemia cells. Blood.

[30] Samuels AL, Heng JY, Beesley AH, Kees UR (2014). Bioenergetic modulation overcomes glucocorticoid resistance in T-lineage acute lymphoblastic leukaemia. Br J Haematol.

[31] Raez LE, Papadopoulos K, Ricart AD, Chiorean EG, Dipaola RS, Stein MN, Rocha Lima CM, Schlesselman JJ, Tolba K, Langmuir VK, Kroll S, Jung DT, Kurtoglu M (2013). A phase I dose-escalation trial of 2-deoxy-D-glucose alone or combined with docetaxel in patients with advanced solid tumors. Cancer Chemother Pharmacol.

[32] Bendell JC, Rodon J, Burris HA, de Jonge M, Verweij J, Birle D, Demanse D, De Buck SS, Ru QC, Peters M, Goldbrunner M, Baselga J (2012). Phase I, dose-escalation study of BKM120, an oral pan-Class I PI3K inhibitor, in patients with advanced solid tumors. J Clin Oncol.

[33] Brown JR, Byrd JC, Coutre SE, Benson DM, Flinn IW, Wagner-Johnston ND, Spurgeon SE, Kahl BS, Bello C, Webb HK, Johnson DM, Peterman S, Li D (2014). Idelalisib, an inhibitor of phosphatidylinositol 3-kinase p110delta, for relapsed/refractory chronic lymphocytic leukemia. Blood.

[34] Mayer IA, Abramson VG, Isakoff SJ, Forero A, Balko JM, Kuba MG, Sanders ME, Yap JT, Van den Abbeele AD, Li Y, Cantley LC, Winer E, Arteaga CL (2014). Stand up to cancer phase Ib study of pan-phosphoinositide-3-kinase inhibitor buparlisib with letrozole in estrogen receptor-positive/human epidermal growth factor receptor 2-negative metastatic breast cancer. J Clin Oncol.

[35] Wilson TR, Fridlyand J, Yan Y, Penuel E, Burton L, Chan E, Peng J, Lin E, Wang Y, Sosman J, Ribas A, Li J, Moffat J (2012). Widespread potential for growth-factor-driven resistance to anticancer kinase inhibitors. Nature.

[36] Neron S, Pelletier A, Chevrier MC, Monier G, Lemieux R, Darveau A (1996). Induction of LFA-1 independent human B cell proliferation and differentiation by binding of CD40 with its ligand. Immunol Invest.

[37] Willimott S, Baou M, Naresh K, Wagner SD (2007). CD154 induces a switch in pro-survival Bcl-2 family members in chronic lymphocytic leukaemia. Br J Haematol.

[38] Willimott S, Baou M, Huf S, Deaglio S, Wagner SD (2007). Regulation of CD38 in proliferating chronic lymphocytic leukemia cells stimulated with CD154 and interleukin-4. Haematologica.

[39] Zaitseva L, Murray MY, Shafat MS, Lawes MJ, MacEwan DJ, Bowles KM, Rushworth SA (2014). Ibrutinib inhibits SDF1/CXCR4 mediated migration in AML. Oncotarget.

[40] Newton R, Holden NS (2007). Separating transrepression and transactivation: a distressing divorce for the glucocorticoid receptor?. Mol Pharmacol.

[41] Jeong JY, Jeoung NH, Park KG, Lee IK (2012). Transcriptional regulation of pyruvate dehydrogenase kinase. Diabetes Metab J.

[42] Kwon HS, Huang B, Unterman TG, Harris RA (2004). Protein kinase B-alpha inhibits human pyruvate dehydrogenase kinase-4 gene induction by dexamethasone through inactivation of FOXO transcription factors. Diabetes.

[43] Puthanveetil P, Wang Y, Wang F, Kim MS, Abrahani A, Rodrigues B (2010). The increase in cardiac pyruvate dehydrogenase kinase-4 after short-term dexamethasone is controlled by an Akt-p38-forkhead box other factor-1 signaling axis. Endocrinology.

[44] Petre-Draviam CE, Williams EB, Burd CJ, Gladden A, Moghadam H, Meller J, Diehl JA, Knudsen KE (2005). A central domain of cyclin D1 mediates nuclear receptor corepressor activity. Oncogene.

