# Supplementary material for: Janus and PI3-kinases mediate glucocorticoid resistance in activated chronic leukemia cells
Source: Oncotarget. 2016 Aug 25;7(45):72608–21. doi: 10.18632/oncotarget.11618 (PMC5341931; doi:10.18632/oncotarget.11618)
Supplement: Supplementary file 1 [file oncotarget-07-72608-s001.pdf]

Janus and PI3-kinases mediate glucocorticoid resistance in activated chronic leukemia cells

Supplementary Material

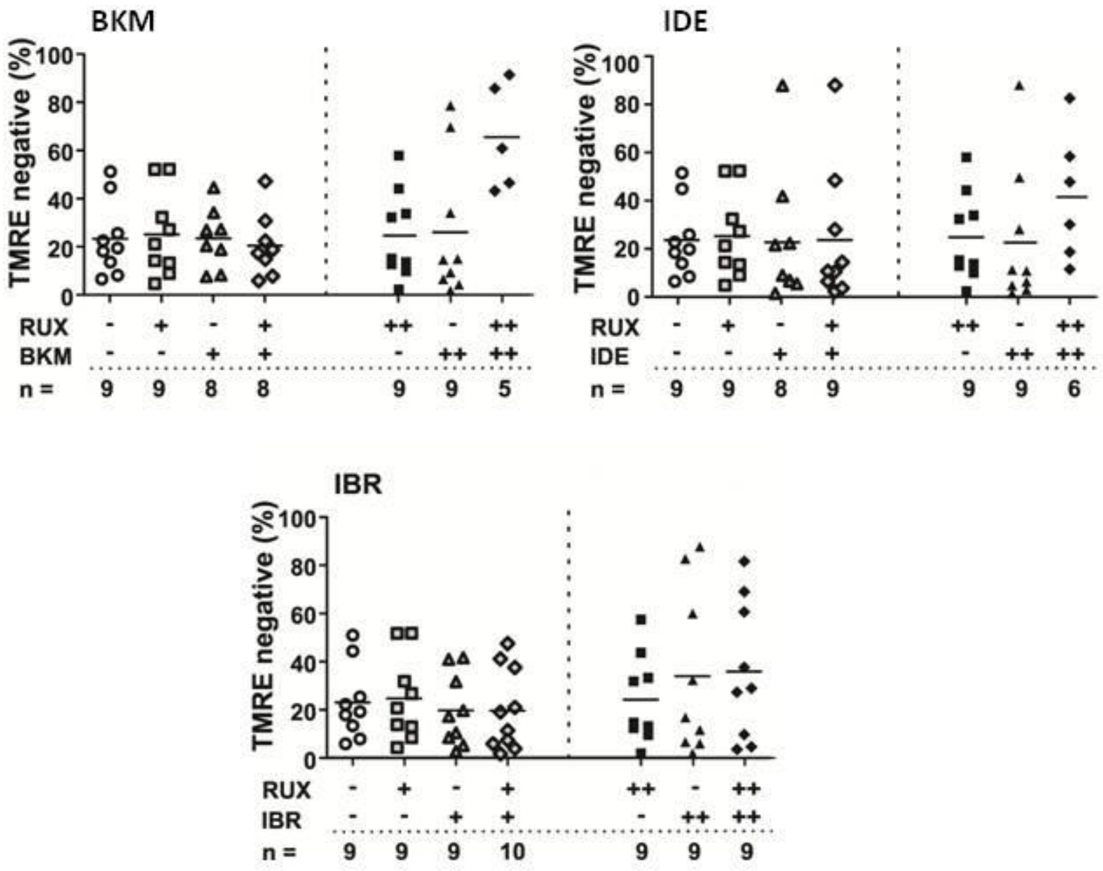

Supplementary Figure 1. Effect of combinations of ruxolitinib and PI3K inhibitors on activated CLL cells without DEX. CLL cells were purified and cultured in the presence of IL-2 and resiquimod (2S) and treated with various concentrations of ruxolitinib (RUX), buparlisib (BKM), idelalisib (IDE), or ibrutinib (IBR) alone or in combination. After 72 h, cells were costained with TMRE to detect mitochondrial membrane potential and Draq5 for cell segmentation and then were imaged using the Opera high-content fluorescent confocal microscope and image analysis software as described in the materials and methods. Cell death was indicated by loss of mitochondrial membrane potential (TMRE negative (%)). The vertical scatter plots show individual results from 2S-stimulated cells from 5-9 patients. Mean value of % of cell death is indicated by the lines. Data points from each patient are shown as percentage average of TMRE negative cells from 8-12 fields of view taken from 2-3 replicate wells for each condition. Low doses of ruxolitinib (100 nM) and PI3K inhibitors BKM or IDE (30 nM each) (indicated by + in the x-axis) did not increase cell death in contrast to combinations of 1 µM RUX and 3 µM PI3K inhibitors (labeled by ++). Low doses of ruxolitinib (100 nM) and ibrutinib (30 nM) (marked as +) did not increase cell death and higher concentrations of 1 µM RUX and 3 µM IBR (marked as ++) only slightly increased cell death (TMRE negative (%)).
