# Supplementary material for: Janus and PI3-kinases mediate glucocorticoid resistance in activated chronic leukemia cells
Source: Oncotarget. 2016 Aug 25;7(45):72608–21. doi: 10.18632/oncotarget.11618 (PMC5341931; doi:10.18632/oncotarget.11618)
Supplement: Supplementary file 2 [file oncotarget-07-72608-s002.docx]

Janus and PI3-kinases mediate glucocorticoid resistance in activated chronic leukemia cells

**Supplementary Tables**

**Supplementary Table S1: Patient Characteristics**

| **Pt. No.^a^** | **Sex** | **Age**  **(yrs)** | **Time**  **(yrs)^b^** | **WBC**  **(x10^9^/L)** | **Stage^c^** | **CD38 (%)** | **β2M^d^** | **FISH** | **Tx^e^** |
| --- | --- | --- | --- | --- | --- | --- | --- | --- | --- |
| 1 | m | 69 | 7 | 189 | 4 | 20 | 4.2 | 13q | 3 |
| 2 | m | 76 | 10 | 152 | 4 | 20 | 8.2 | 13q | 3 |
| 3 | m | 64 | 3 | 252 | 3 | 48 | 5.1 | normal | 2 |
| 4 | m | 79 | 3 | 109 | 2 | 1 | 3.3 | 13q | 0 |
| 5 | m | 66 | 10 | 102 | 2 | 1 | 2.8 | 13q | 1 |
| 6 | m | 51 | 2 | 111 | 2 | 1 | 2.8 | 13q | 1 |
| 7 | m | 84 | 4 | 138 | 3 | 22 | 4.3 | 17p | 2 |
| 8 | f | 77 | 4 | 244 | 4 | 4 | 6.4 | 13q | 1 |
| 9 | f | 38 | 5 | 322 | 3 | 1 | 2.9 | 13q | 1 |
| 10 | m | 76 | 5 | 188 | 4 | 3 | 7 | na | 0 |
| 11 | m | 64 | 7 | 222 | 3 | 15 | 3.7 | T12 | 1 |
| 12 | m | 64 | 4 | 106 | 4 | 1 | 2.9 | 13q | 1 |
| 13 | f | 76 | 7 | 139 | 3 | 4 | 4 | 13q | 1 |
| 14 | m | 57 | 7 | 151 | 3 | 12 | 2.6 | normal | 1 |
| 15 | m | 69 | 7 | 121 | 4 | 7 | 3.5 | 13q | 3 |
| 16 | m | 89 | 4 | 225 | 4 | 22 | 6.4 | 13q | 1 |
| 17 | f | 69 | 8 | 239 | 3 | 7 | 4.8 | 13q | 1 |
| 18 | f | 86 | 5 | 87 | 1 | 2 | 2.8 | na | 0 |
| 19 | m | 69 | 10 | 111 | 4 | 3 | 1.9 | na | 2 |
| 20 | m | 69 | 10 | 60 | 4 | 1 | 2.5 | 13q | 2 |
| 21 | f | 78 | 12 | 203 | 3 | 9 | 3.1 | 13q | 0 |
| 22 | m | 58 | 4 | 264 | 4 | 3 | 4 | 13q | 1 |
| 23 | m | 66 | 8 | 210 | 2 | 1 | 1.8 | na | 0 |
| 24 | m | 67 | 5 | 56 | 1 | 11 | 1.9 | na | 0 |
| 25 | f | 70 | 5 | 102 | 2 | 1 | 2.7 | na | 0 |
| 26 | m | 79 | 6 | 120 | 1 | 44 | 2.9 | na | 0 |
| 27 | f | 67 | 7 | 78 | 3 | 1 | 3.1 | na | 0 |
| 28 | f | 64 | 5 | 21 | 1 | 1 | 2.6 | na | 0 |
| 29 | f | 78 | 3 | 246 | 4 | 25 | 10.1 | 17p | 2 |
| 30 | m | 69 | 2 | 105 | 4 | 15 | 3.8 | normal | 1 |
| 31 | m | 77 | 13 | 21 | 4 | 2 | 4 | t12 | 2 |
| 32 | m | 56 | 3 | 155 | 4 | 1 | 3.5 | 13q | 0 |
| 33 | f | 74 | 5 | 150 | 3 | 1 | 5.6 | 13q | 2 |
| 34 | f | 88 | 19 | 20 | 4 | 5 | 16 | t12 | 2 |
| 35 | f | 83 | 12 | 82 | 1 | 1 | 4.7 | normal | 0 |
| 36 | f | 61 | 22 | 56 | 4 | 1 | 2.4 | 13q | 0 |
| 37 | m | 83 | 21 | 201 | 4 | 1 | 4 | 11q | 2 |
| 38 | f | 81 | 5 | 202 | 4 | 20 | 5 | normal | 2 |
| 39 | f | 62 | 7 | 61 | 2 | 1 | 2.3 | na | 0 |
| 40 | m | 62 | 3 | 100 | 3 | 1 | 3.2 | na | 0 |
| 41 | f | 73 | 11 | 107 | 3 | 1 | 5.1 | na | 0 |
| 42 | m | 63 | 11 | 464 | 4 | 1 | 3.3 | na | 0 |

a. Corresponding patient numbers are maintained throughout this article.

b. Time since diagnosis

c. Rai stage 0=lymphocytosis; I= with adenopathy; II= with hepatosplenomegaly; III= with anemia; IV= with thromocytopenia.

d. Normal range is 0.6-2.3 μg/ml

e. Including alkylator-, fludarabine-, high dose glucocorticoid- and/or antibody-based treatments, splenectomy, radiation, and Ruxolitinib

na=not available

**Supplementary Table S2**

320 KIs were screened for DEX-enhanced-toxicity against 2S-stimulated CLL cells derived from 3 patients. Compound number (No.) corresponds to compound numbers indicated in Figure 2. Compounds that are licensed are indicated as ‘FDA approved’, compounds that are currently used in clinical trials (phase I-III) are indicated as ‘in clinical trial’. All other compounds are indicated as ‘preclinical’. The first 38 inhibitors (bold) were identified as most common Hit compounds with a z-score above 3.

| **No.** | **Compound** | **FDA status** |
| --- | --- | --- |
| **1** | **K-252a, K-2151** | preclinical |
| **2** | **NSC 663284** | preclinical |
| **3** | **ZM 336372** | preclinical |
| **4** | **AMG-Tie2-1** | in clinical trial |
| **5** | **AT-7519** | preclinical |
| **6** | **KU-55933** | preclinical |
| **7** | **Akt-I-1** | preclinical |
| **8** | **Merck-5, Mk-5** | preclinical |
| **9** | **PD 184161** | preclinical |
| **10** | **GSK690693** | preclinical |
| **11** | **CP-724714** | preclinical |
| **12** | **BMS-599626, AC-480** | in clinical trial |
| **13** | **PHA690509** | preclinical |
| **14** | **Arcyriaflavin A** | preclinical |
| **15** | **PF-02341066, PF-2341066** | preclinical |
| **16** | **DCA, Dichloroacetate** | **FDA approved** |
| **17** | **MK-2206** | preclinical |
| **18** | **BAY 61-3606 hydrochloride, sc-202351** | preclinical |
| **19** | **Genistein** | preclinical |
| **20** | **TWS119** | preclinical |
| **21** | **PV-1019, NSC-744039** | preclinical |
| **22** | **PD 198306** | preclinical |
| **23** | **AR-A014418** | preclinical |
| **24** | **ZM 323881 hydrochloride** | preclinical |
| **25** | **PD04217903, PF-04217903** | preclinical |
| **26** | **AP-24534** | preclinical |
| **27** | **AG 18, RG-50810, TyrphostinA23** | preclinical |
| **28** | **LY 333531 mesylate, Ruboxistaurin** | in clinical trial |
| **29** | **AZD-7762 hydrochloride** | in clinical trial |
| **30** | **Imatinib (free base), Gleevec, Glivec, CGP-57148B, STI-571** | **FDA approved** |
| **31** | **Axitinib, AG-013736** | **FDA approved** |
| **32** | **BX 795** | preclinical |
| **33** | **TAK-715** | preclinical |
| **34** | **API-2, Triciribine, NSC154020, TCN** | preclinical |
| **35** | **CGP 57380** | preclinical |
| **36** | **PIM1/2 Kinase Inhibitor VI** | preclinical |
| **37** | **PD173955** | preclinical |
| **38** | **Ryuvidine** | preclinical |
| 39 | SU-11274, PKI-SU11274 | preclinical |
| 40 | STO-609 acetic acid | preclinical |
| 41 | NVP-AEW541, AEW541 | preclinical |
| 42 | K-252c, Staurosporine Aglycone | preclinical |
| 43 | HDS 029 | preclinical |
| 44 | PF562271 | preclinical |
| 45 | IKK 16 | preclinical |
| 46 | Janex-1, WHI-P131 | preclinical |
| 47 | JNJ-38877605 | in clinical trial |
| 48 | AZD6244, ARRY-142886 | in clinical trial |
| 49 | BMS-345541 | preclinical |
| 50 | AV951, KRN951, Tivozanib | **FDA approved** |
| 51 | BIBU 1361 dihydrochloride | preclinical |
| 52 | Wortmannin, KY 12420 | preclinical |
| 53 | SKF-86002 | preclinical |
| 54 | BMS-2 | preclinical |
| 55 | CGP-74514A hydrochloride | preclinical |
| 56 | AG 494 | preclinical |
| 57 | EO 1428 | preclinical |
| 58 | Dioctanoylglycol | preclinical |
| 59 | PKC-412, CGP41251, Midostaurin | in clinical trial |
| 60 | R1487 | preclinical |
| 61 | Bisindoylmaleimide X, HCl salt | preclinical |
| 62 | BIBX 1382 dihydrochloride, Falnidamol | preclinical |
| 63 | IMD 0354 | preclinical |
| 64 | BIBF-1120, Intedanib, Vargatef | **FDA approved** |
| 65 | Compound 52, NG-52 | preclinical |
| 66 | PQ401 | preclinical |
| 67 | LY-294002 | preclinical |
| 68 | AG13958 | preclinical |
| 69 | Arctigenin, (-)-Arctigenin | preclinical |
| 70 | Bosutinib, SKI-606 | **FDA approved** |
| 71 | MLN-8237 | preclinical |
| 72 | GSK269962A | preclinical |
| 73 | Ki 8751 | preclinical |
| 74 | IPA 3 | preclinical |
| 75 | Sorafenib,p-Toluenesulfonate Salt, Bay 43-9006, Nexavar | **FDA approved** |
| 76 | FAK Inhibitor 14 | preclinical |
| 77 | Brivanib, BMS-540215 | **FDA approved** |
| 78 | FPA 124 | preclinical |
| 79 | SB 218078 | preclinical |
| 80 | Pimecrolimus, Elidel, SDZ-ASM-981 | **FDA approved** |
| 81 | PHA 767491 hydrochloride | preclinical |
| 82 | SU 5402 | preclinical |
| 83 | SB 239063 | preclinical |
| 84 | Lestaurtinib, CEP-701, KT-5555, SPM-924 | in clinical trial |
| 85 | EKI-785, CL-387,785 | preclinical |
| 86 | Bisindolylmaleimide I hydrochloride, GF 109203X HCl, Go 6850 HCl | preclinical |
| 87 | SGX-523 | preclinical |
| 88 | PI-93 | preclinical |
| 89 | KN-62 | preclinical |
| 90 | AT9283 | preclinical |
| 91 | Pazopanib hydrochloride, GW-786034, Armala, Votrient | **FDA approved** |
| 92 | GSK 650394 | preclinical |
| 93 | GSK-461364, GSK461364 | in clinical trial |
| 94 | A-443654 | preclinical |
| 95 | SU 16f | preclinical |
| 96 | TCS 2312 dihydrochloride | preclinical |
| 97 | GDC-0879, AR-00341677 | preclinical |
| 98 | PIM 1 Inhibitor 2 | preclinical |
| 99 | BI-D1870 | preclinical |
| 100 | CGP 57380 | preclinical |
| 101 | BMS-5 | preclinical |
| 102 | Roscovitine, CYC202, Seliciclib | in clinical trial |
| 103 | HA 1100 hydrochloride, Hydroxyfasudil | preclinical |
| 104 | H 1152, Glycyl dihydrochloride | preclinical |
| 105 | AEG 3482 | preclinical |
| 106 | PP-1, PP1, AGL-1872, Tyrphostin-PP1 | preclinical |
| 107 | Alvocidib, HMR-1275, L-868275, MDL-107826A, NSC-649890, L-868276, Flavopiridol | in clinical trial |
| 108 | Sal003 | preclinical |
| 109 | PIK-294 | preclinical |
| 110 | PD 98059 | preclinical |
| 111 | XL-147, XL147, SAR-245408 | preclinical |
| 112 | SP-600125 | preclinical |
| 113 | TPL2 | preclinical |
| 114 | C-1 | preclinical |
| 115 | BIBW-2992, Tovok | in clinical trial |
| 116 | Rapamycin, Sirolimus, Rapamune | **FDA approved** |
| 117 | BMS-3 | preclinical |
| 118 | Purvalanol B, NG 95 | preclinical |
| 119 | SNS-314 | preclinical |
| 120 | Purvalanol A, NG 60 | preclinical |
| 121 | PD0325901 | preclinical |
| 122 | Masitinib mesylate, AB1010 | **FDA approved** |
| 123 | PHA-739358, Danusertib | in clinical trial |
| 124 | Go 6976, PD 406976 | preclinical |
| 125 | GW8510 | preclinical |
| 126 | L 779450, Raf Kinase Inhibitor IV | preclinical |
| 127 | PF 573228 | preclinical |
| 128 | XRP44X | preclinical |
| 129 | Sorafenib, Bay 43-9006, Nexavar | **FDA approved** |
| 130 | AC220 | preclinical |
| 131 | Nilotinib, Tasigna, AMN-107 | **FDA approved** |
| 132 | AV-412, MP-412 | preclinical |
| 133 | CC-401 | preclinical |
| 134 | IC87114, PIK-39, PIK-23 | preclinical |
| 135 | 2-(p-Hydroxyanilino)-4-(p-chlorophenyl) thiazole, HCl | preclinical |
| 136 | RWJ 67657 | preclinical |
| 137 | SL327 | preclinical |
| 138 | CT 99021, CHIR 99021 | preclinical |
| 139 | 5-(3-Methoxy-4-((4-methoxybenzyl)oxy)benzyl)-pyrimidine-2,4-diamine | preclinical |
| 140 | Imidazolo-oxindole PKR inhibitor C16 | preclinical |
| 141 | Cyclopolin9 | preclinical |
| 142 | Cediranib, AZD-2171, Recentin | **FDA approved** |
| 143 | CYC-116 | preclinical |
| 144 | Olomoucine | preclinica |
| 145 | PIK 90 | preclinical |
| 146 | PD173074 | preclinical |
| 147 | Ro 31-8220 mesylate, Bisindolylmaleimide IX | preclinical |
| 148 | PP2, AG1879 | preclinical |
| 149 | SU 6656 | preclinical |
| 150 | SKF 86002 dihydrochloride | preclinical |
| 151 | MK-1775, MK1775 | in clinical trial |
| 152 | TCS PIM-1 1 | preclinical |
| 153 | GW2974 | preclinical |
| 154 | PD173955-Analogue 1 | preclinical |
| 155 | Tyrphostin AG 1296 | preclinical |
| 156 | NSC 109555 ditosylate, DDUG | preclinical |
| 157 | Akt-I-1,2 | preclinical |
| 158 | AZD-1152-HQPA, Barasertib | In clinical trial |
| 159 | Staurosporine | preclinical |
| 160 | NU 2058 | preclinical |
| 161 | AZD-1152, Barasertib | **FDA approved** |
| 162 | YM201636 | preclinical |
| 163 | Rho Kinase Inhibitor V | preclinical |
| 164 | SKI II | preclinical |
| 165 | SB242235 | preclinical |
| 166 | SU 4312, DMBI | preclinical |
| 167 | GSK-1904529A, GSK1904529A | in clinical trial |
| 168 | Compound 401 | preclinical |
| 169 | IRAK-1/4 Inhibitor I | preclinical |
| 170 | SGI-1776 | preclinical |
| 171 | Alsterpaullone | preclinical |
| 172 | PD-0332991, PD-332991 | in clinical trial |
| 173 | Akt 1/2 Kinase inhibitor | preclinical |
| 174 | Necrostatin-1 | preclinical |
| 175 | PS1145 dihydrochloride | preclinical |
| 176 | U0126 | preclinical |
| 177 | SR3677 | preclinical |
| 178 | Tyrphostin AG 1478 mesylate | preclinical |
| 179 | CP-690550 | preclinical |
| 180 | SB 202190, FHPI | preclinical |
| 181 | RHO-15 | preclinical |
| 182 | Rottlerin | preclinical |
| 183 | VX-702, VX-850, VX-954, KVK-702 | in clinical trial |
| 184 | ZM447439 | preclinical |
| 185 | ERK2 inhibitor | preclinical |
| 186 | Src I1 | preclinical |
| 187 | PI-103 | preclinical |
| 188 | SNS-032, BMS387032 | in clinical trial |
| 189 | AG 825, Tyrphostin AG 825 | preclinical |
| 190 | Tyrphostin B44, (-) enantiomer | preclinical |
| 191 | ABT-869, Linifanib, AL-39324, RG-3635 | **FDA approved** |
| 192 | Erlotinib HCl, CP-358774, OSI-774, Tarceva, NSC-718781, RG-1415, Ro-50-8231 | **FDA approved** |
| 193 | CI-1033, Canertinib, PD-183805, SN-26606 | in clinical trial |
| 194 | Tyrphostin SU 1498, SU 1498 | preclinical |
| 195 | PD 184352, CI-1040 | in clinical trial |
| 196 | Sphingosine kinase Inhibitor 2 | preclinical |
| 197 | PD 158780 | preclinical |
| 198 | ABT 702 dihydrochloride | preclinical |
| 199 | GW583340 dihydrochloride | preclinical |
| 200 | Dasatinib, BMS-354825, Sprycel, NSC-732517 | **FDA approved** |
| 201 | A 83-01 | preclinical |
| 202 | AG 490, TyrphostinAG 490 | preclinical |
| 203 | SB 415286 | preclinical |
| 204 | SU 5416, Semaxinib | in clinical trial |
| 205 | TX-1918 | preclinical |
| 206 | AS 252424 | preclinical |
| 207 | AZD0530, Saracatinib, NSC-735464 | **FDA approved** |
| 208 | BI 2536 | preclinical |
| 209 | SU 9516 | preclinical |
| 210 | BI 78D3 | preclinical |
| 211 | MP-470 | preclinical |
| 212 | RDEA-119, AR-119 | in clinical trial |
| 213 | TBB, NSC 231634 | preclinical |
| 214 | NSC 693868 | preclinical |
| 215 | TCS JNK 5a | preclinical |
| 216 | 2-Dimethylamino-4,5,6,7-tetrabromo-1H-benzimidazole, DMAT, Casein Kinase II | preclinical |
| 217 | Sunitinib Malate, Sutent, SU-11248, PHA-290940AD | **FDA approved** |
| 218 | NU6102 | preclinical |
| 219 | PP3 | preclinical |
| 220 | AMG-47a | in clinical trial |
| 221 | NH125 | preclinical |
| 222 | Lim2 Kinase Inhibitor | preclinical |
| 223 | 1-Naphthyl PP1, 1-NA-PP 1 | preclinical |
| 224 | KU0063794 | preclinical |
| 225 | AZ-960 | preclinical |
| 226 | BX912 | preclinical |
| 227 | Imatinib Mesylate, Gleevec, Glivec, CGP-57148B, STI-571 | **FDA approved** |
| 228 | AG 213, Tyrphostin AG 213 | preclinical |
| 229 | SD 208 | preclinical |
| 230 | SD-06 | preclinical |
| 231 | D4476 | preclinical |
| 232 | JNJ-10198409, RWJ 540973 | preclinical |
| 233 | Ellagic acid | preclinical |
| 234 | BMS-536924 | preclinical |
| 235 | OSU-03012 hydrochloride | preclinical |
| 236 | Kenpaullone, alsterpolone | preclinical |
| 237 | TAE-684 | preclinical |
| 238 | Vatalanib dihydrochloride, PTK-787, ZK-222584, CGP79787D | **FDA approved** |
| 239 | Temsirolimus, CCI-779, Torisel | **FDA approved** |
| 240 | SIS3 | preclinical |
| 241 | TGX 221 | preclinical |
| 242 | DMPQ dihydrochloride | preclinical |
| 243 | JX 401 | preclinical |
| 244 | CID 755673 | preclinical |
| 245 | PD 153035, AG 1517, Compound 32, SU 5271, ZM 252868 | preclinical |
| 246 | Y-27632 | preclinical |
| 247 | Motesanib diphosphate salt, AMG-706 | in clinical trial |
| 248 | LY-364947, HTS 466284 | preclinical |
| 249 | GW441756 hydrochloride | preclinical |
| 250 | IC261, SU5607 | preclinical |
| 251 | FK-506, Tacrolimus, Fujimycin, Prograf | **FDA approved** |
| 252 | Lapatinib ditosylate, Tykerb, GW572016 | **FDA approved** |
| 253 | NU7026, LY293646 | preclinical |
| 254 | JNJ 28871063 hydrochloride | preclinical |
| 255 | 10-DEBC hydrochloride | preclinical |
| 256 | PD 180970 | preclinical |
| 257 | Fasudil HCl, AT-877, HA-1077 (diHCl), Eril | preclinical |
| 258 | BI-6727, volasertib | in clinical trial |
| 259 | TCS 359 | preclinical |
| 260 | CGK 733 | preclinical |
| 261 | CYT11387, CYT387 | preclinical |
| 262 | SU 6668 | preclinical |
| 263 | 5-Iodotubercidin | preclinical |
| 264 | Quercetin | preclinical |
| 265 | AS604850 | preclinical |
| 266 | ML 9 hydrochloride | preclinical |
| 267 | LFM-A13 | preclinical |
| 268 | R 59-022 | preclinical |
| 269 | GW-5074 | preclinical |
| 270 | H 89 dihydrochloride | preclinical |
| 271 | NVP-BEZ235, BEZ235 | in clinical trial |
| 272 | 6-bromoindirubin-3'-oxime, BIO | preclinical |
| 273 | Dorsomorphin dihydrochloride, BML-275 | preclinical |
| 274 | AS 601245 | preclinical |
| 275 | XL-880, GSK-1363089, EXEL-2880, GSK-089, foretinib | **FDA approved** |
| 276 | GTP 14564 | preclinical |
| 277 | JNJ-7706621 | preclinical |
| 278 | PIK-75 | preclinical |
| 279 | PD 407824 | preclinical |
| 280 | Dovitinib, CHIR-258, TKI-258, GFKI-258 | **FDA approved** |
| 281 | Lavendustin A, RG 14355 | preclinical |
| 282 | GW 843682X | preclinical |
| 283 | Gefitinib, Iressa, ZD1839 | **FDA approved** |
| 284 | 6-[4-(2-Piperidin-1-ylethoxy)phenyl]-3-pyridin-4-ylpyrazolo[1,5-a]pyrimidine | preclinical |
| 285 | Enzastaurin, LY-317615 | in clinical trial |
| 286 | ZM 39923 hydrochloride | preclinical |
| 287 | SB-505124 hydrochloride hydrate | preclinical |
| 288 | SB590885 | preclinical |
| 289 | PF 670462 | preclinical |
| 290 | PI-828 | preclinical |
| 291 | PHA 665752 | preclinical |
| 292 | VX-680, MK-0457, Tozasertib, VX6 | in clinical trial |
| 293 | NSC 625987 | preclinical |
| 294 | SB 203580 | preclinical |
| 295 | SB 431542 | preclinical |
| 296 | 7-Cyclopentyl-5-(4-phenoxyphenyl)-7H-pyrrolo[2,3-d]pyrimidin-4-ylamine | preclinical |
| 297 | N-(4-Pyridyl)-N'-(2,4,6-trichlorophenyl)urea, Rho Kinase Inhibitor II | preclinical |
| 298 | CHIR 98014 isomer | preclinical |
| 299 | Chelerythrine chloride | preclinical |
| 300 | ZSTK474 | preclinical |
| 301 | PD 169316 | preclinical |
| 302 | SB 216763 | preclinical |
| 303 | MLN-518, CT 53518, Tandutinib | in clinical trial |
| 304 | GDC-0941 bismesylate | preclinical |
| 305 | (5Z)-7-Oxozeaenol | preclinical |
| 306 | ER 27319 maleate | preclinical |
| 307 | TPCA-1 | preclinical |
| 308 | Mubritinib, TAK-165, D04025 | **FDA approved** |
| 309 | 2-Thio(3-iodobenzyl)-5-(1-pyridyl)-[1,3,4]-oxadiazole | preclinical |
| 310 | Everolimus, RAD-001, Certican | **FDA approved** l |
| 311 | Vandetanib, Zactima, ZD6474, AZD-6474 | **FDA approved** |
| 312 | ZM 306416 hydrochloride | **FDA approved** |
| 313 | WHI-P 154 | preclinical |
| 314 | SC 514 | preclinical |
| 315 | Chk2 Inhibitor II | preclinical |
| 316 | PLX4720, Raf Kinase Inhibitor V | preclinical |
| 317 | SD 169 | preclinical |
| 318 | PF-04217903 | preclinical |
| 319 | E7080 | preclinical |
| 320 | Ki20227 (+/-) | in clinical trial |
